# Incidence of New-Onset Inflammatory Bowel Disease, Oral and Gastrointestinal Candidiasis, Herpes Zoster, Pulmonary Tuberculosis, and Major Cardiovascular Events in Patients with Moderate-to-Severe Psoriasis Exposed to Biologics

**DOI:** 10.3390/jcm12247653

**Published:** 2023-12-13

**Authors:** Da-Hyun Kang, Bark-Lynn Lew, Soon-Hyo Kwon

**Affiliations:** Department of Dermatology, Kyung Hee University Hospital at Gangdong, Kyung Hee University College of Medicine, Seoul 05278, Republic of Korea; jenny967@naver.com (D.-H.K.); bellotte@hanmail.net (B.-L.L.)

**Keywords:** adverse events, biologics, candidiasis, herpes zoster, inflammatory bowel disease, MACEs, psoriasis, tuberculosis

## Abstract

The multicenter, retrospective cohort study was aimed at examining adverse events in biologic-treated patients with moderate-to-severe psoriasis by using a real-world database. Thus, we analyzed exposure-adjusted incidence rates for new-onset inflammatory bowel disease (IBD), oral and gastrointestinal candidiasis, pulmonary tuberculosis, herpes zoster, and major cardiovascular events (MACEs) in biologic-treated patients with moderate-to-severe psoriasis. Overall, 2085 patients were found to have been exposed to tumor necrosis factor (TNF)-α, interleukin (IL)-12/23, IL-17, and IL-23 inhibitors (*n* = 463, 540, 635, and 447, respectively). No patient developed new-onset IBD. The incidence rates of oral and gastrointestinal candidiasis were comparable between patients treated with IL-23 and IL-17 inhibitors (5.6 and 5.3 per 1000 PY, respectively). None treated with IL-17 or IL-23 inhibitors reported pulmonary tuberculosis. The incidence rate of herpes zoster was the highest in patients treated with TNF-α inhibitors (17.0 per 1000 PY), followed by IL-17, IL-23, and IL-12/23 inhibitors (13.3, 7.8, and 2.7 per 1000 PY, respectively). MACEs were not reported in patients treated with IL-17 inhibitors but were reported in those treated with TNF-α, IL-23, and IL-12/23 inhibitors (incidence: 5.6, 3.8, and 1.8 per 1000 PY, respectively). The study indicated favorable safety profiles of biologics in Korean patients with moderate-to-severe psoriasis.

## 1. Introduction

Psoriasis is a type of chronic inflammatory dermatosis that leads to the deterioration of the affected patients’ quality of life [1]. The first biologics for treating psoriasis were tumor necrosis factor (TNF)-α inhibitors, which target TNF-α from Th1 cells [2]. After that, the role of Th17 cells in the pathogenesis of psoriasis was identified, and interleukin (IL)-12/23 inhibitors targeting the p40 subunit of IL-12 and IL-23, which induce the differentiation of naïve T cells into Th1 and Th17 cells, respectively, were developed [3,4]. Cumulative data from clinical trials and real-world studies have demonstrated the high efficacy and favorable safety profiles of biologics, especially IL-17 and IL-23 inhibitors, for treating moderate-to-severe plaque psoriasis [5,6,7].

Considerations for selecting biologics for patients with psoriasis involve the severity of the disease, affected area, presence of psoriatic arthritis (PsA), or other comorbidities such as inflammatory bowel disease (IBD), infection risk, and CVD risk [7]. In particular, IL-17 inhibitors have been reported to be associated with the aggravation of IBD and an increased risk of oral or gastrointestinal (GI) candidiasis [8]. Improvement of vascular flow was noted following the administration of an IL-17 inhibitor in patients with psoriasis, suggesting its potential for preventing CVD [9]. However, the risk of new-onset IBD in patients with psoriasis following the administration of IL-17 inhibitors has not yet been elucidated. Furthermore, the comparative risk of oral/GI candidiasis or CVD between IL-17 inhibitors and other biologics in patients with moderate-to-severe psoriasis has not been evaluated.

The present study was aimed at examining the adverse events in patients with moderate-to-severe psoriasis who were treated with currently approved biologics in Korea by using the Observational Outcomes Partnership-Common Data Model (OMOP-CDM) of large-scale real-world data validated in previous studies. Our purpose was to elucidate the real-world incidences of adverse events of interest, including new-onset IBD, oral/GI candidiasis, pulmonary tuberculosis, herpes zoster, and major cardiovascular events (MACEs) in patients with psoriasis treated using TNF-α, IL-12/23, IL-17, and IL-23 inhibitors.

## 2. Materials and Methods

### 2.1. Data Sources

A multicenter, retrospective cohort study was performed by acquiring real-world clinical data from 19,321,164 patients from 16 hospitals in Korea. De-identified patient-level electronic health record data were converted to the OMOP-CDM ver. 5.3.1. Variable terms used in the electronic health records of each database were standardized into standard terms (concepts) using mapping. For example, psoriasis (International Classification of Diseases [ICD]-10 code L40) is converted into standard concept ID #140168. Standard terms and matched concept IDs can be found at https://athena.ohdsi.org/search-terms/start (accessed on 13 October 2023). OMOP-CDM databases were provided to researchers who were affiliated with collaborating organizations for distributed network analysis as an open source using FEEDER-NET (https://feedernet.com/dataMap, accessed on 13 October 2023), a Korean health data platform based on OMOP-CDM [10].

The following 16 databases were included in our study: database version; number of patients in the database; time period of the database; (1) Kangdong Sacred Heart Hospital (KDH_5.3.1_04; 1,194,685 patients; November 1986–September 2022), (2) Kyung Hee University Hospital at Gangdong (KHNMC_5.3.0_03; 805,332 patients; June 2006–October 2021), (3) Ewha Womens University Medical Center (EUMC_5.3.1_02; 1,667,671 patients; January 2021–December 2021), (4) Pusan National University Hospital (PNUH_5.3.0_02; 1,753,001 patients; February 2011–August 2019), (5) Ajou University Medical Center (AUMC_5.3.1_06; 2,873,443 patients; January 1994–February 2022), (6) Kangwon National University Hospital (KWMC_5.3.1_04; 567,439 patients; January 2003–January 2022), (7) Kyung Hee University Medical Center (KHMC_5.3.1_03; 1,168,640 patients; January 2008–February 2022), (8) Wonkwang University Hospital (WKUH_5.3.0_03; 837,461 patients; March 1998–November 2021), (9) Daegu Catholic University Medical Center (DCMC_5.3.1_02; 906,587 patients; January 2005–October 2021), (10) Soonchunhyang University Seoul Hospital (SCHSU_5.3.1_02; 1,098,041 patients; May 2003–May 2021), (11) Soonchunhyang University Bucheon Hospital (SCHBC_5.3.1_02; 1,301,117 patients; February 2001–May 2021), (12) Soonchunhyang University Cheonan Hospital (SCHCA_5.3.1_02; 987,701 patients; June 2006–May 2021), (13) Myongji Hospital (MJH_5.3.0_02; 882,646 patients; September 2003–August 2021), (14) Gyeongsang National University Hospital Changwon (GNUCH_5.3.1_01; 286,642 patients; February 2016–January 2021), (15) Gyeongsang National University Hospital (GNUH_5.3.1_02; 626,663 patients; October 2009–April 2022), and (16) Chungnam National University Hospital (CHNUH_5.3.1_01; 873,511 patients; January 2012–December 2021).

This study was approved by the institutional review board (IRB) of Kyung Hee University Hospital in Gangdong (IRB No. KHNMC 2023-06-004).

### 2.2. Study Design

The target cohort was defined as patients who (1) had the diagnosis of psoriasis (concept ID #140168), (2) were prescribed at least one injection of the study drug for the first time, and (3) underwent a continuous observational period of >30 days before the first prescription. The study drugs include biologics that were approved for the treatment of moderate-to-severe psoriasis in Korea: TNF-α inhibitors (infliximab, concept ID #937368; etanercept, concept ID #1151789; adalimumab, concept ID #1119119), IL-12/23 inhibitors (ustekinumab, concept ID #40161532), IL-17 inhibitors (secukinumab, concept ID #45892883; ixekizumab, concept ID #35603563), and IL-23 inhibitors (guselkumab, concept ID #1593700; risankizumab, concept ID #1511348). The National Health Insurance Service in Korea defines moderate-to-severe psoriasis as the patients who (1) were diagnosed to have psoriasis by a board-certified dermatologist through histopathologic examination; (2) underwent two or more therapies, including methotrexate, cyclosporine, acitretin, and narrowband ultraviolet radiation for at least 24 previous weeks; and (3) had psoriasis area and severity index ≥10 and affected body surface area ≥10% at the time of administration of biologics.

The index date was defined as the day on which the study drug was first prescribed. The cohort exit date was defined as the cessation of the biological therapy, loss of follow-up, or switch to other biologics of a different class, whichever occurred first. The time at risk was set as the time between the index and cohort exit dates.

The primary outcome was the incidence of adverse events in the target cohort. Adverse events of interest were new-onset IBD (concept ID #4074815), oral/GI candidiasis (concept ID #29735, #28974, and #193133), pulmonary tuberculosis (concept ID #253954), herpes zoster (concept ID #443943), and MACEs including acute myocardial infarction (concept ID #312327), stroke (concept ID #443454), and unstable angina (concept ID #321318).

Comorbid diseases were defined as follows: PsA (concept ID #40319772), hypertension (concept ID #320128), type 2 diabetes mellitus (T2DM, concept ID #201826), and hyperlipidemia (concept ID #432867).

### 2.3. Statistical Analysis

ATLAS ver. 2.7.2, an analysis platform embedded in the OMOP-CDM, was used to collect time at risk (person–years, PY), the number of incident cases, and the exposure-adjusted incidence rate (EAIR), defined as the number of cases divided by the time at risk.

## 3. Results

Overall, 2085 patients with moderate-to-severe psoriasis treated with biologics were included in the study. The mean age was 45.6 ± 13.4 years. The male-to-female ratio was 1.86. On the index date, 23.8% of the patients were biologics-experienced, and 18.9% had PsA. The comorbidities reported were as follows: hypertension (9.9%), T2DM (6.6%), hyperlipidemia (7.8%), and IBD (1.1%). Thirty patients (1.4%) had a history of MACEs at the index date.

Table 1 shows the baseline demographic characteristics of the study patients treated with TNF-α, IL-12/23, IL-17, and IL-23 inhibitors (*n* = 463, 540, 635, and 447, respectively). The mean age of the patients treated with IL-23 inhibitors was the highest (48.7 ± 13.9 years), followed by the ages of those treated with IL-17, IL-12/23, and TNF-α inhibitors (46.7 ± 13.3, 44.8 ± 13.2, and 42.1 ± 13.1 years, respectively). The proportion of biologic-experienced patients was the highest in the group treated with IL-23 inhibitors (31.8%), followed by the groups treated with IL-17, IL-12/23, and TNF-α inhibitors (29.8%, 17.6%, and 15.1%, respectively). PsA was present in 39.7% of the patients treated with TNF-α inhibitors, which was the highest among all the groups treated with biologics. The percentages in the other groups were as follows: IL-17 inhibitors, 17.8%; IL12/23 inhibitors, 13.0%; and IL-23 inhibitors, 6.3%. Intergroup differences in the proportions of the patients with comorbid hypertension, T2DM, hyperlipidemia, and a history of MACEs were not numerically different, except in the case of patients with comorbid IBD. The TNF-α inhibitor group had the highest proportion of patients with comorbid IBD (3.7%). No patient with psoriasis or IBD was treated with IL-17 inhibitors.

Table 2 shows the number of cases and EAIRs of the adverse events of interest. No patient newly developed IBD during biological therapy. Oral/GI candidiasis developed in three patients treated with IL-23 inhibitors (EAIR, 5.6 per 1000 PY), four patients treated with IL-17 inhibitors (EAIR, 5.3 per 1000 PY), and one patient treated with IL-12/23 inhibitors (EAIR, 0.9 per 1000 PY). The EAIR of oral/GI candidiasis in patients treated with IL-17 inhibitors was comparable to that in those treated with IL-23 inhibitors. No patient treated with TNF-α inhibitors reported oral/GI candidiasis.

One patient treated with TNF-α and IL-12/23 inhibitors developed pulmonary tuberculosis (EAIR, 1.9 and 0.9 per 1000 PY, respectively). However, no patient treated with IL-17 or IL-23 inhibitors reported pulmonary tuberculosis. The EAIR of herpes zoster was the highest in the TNF-α inhibitor group (17.0 per 1000 PY), followed by the IL-17, IL-23, and IL-12/23 inhibitors (13.3, 7.8, and 2.7 per 1000 PY, respectively). MACE occurred in three patients treated with TNF-α inhibitors (EAIR, 5.6 per 1000 PY), two patients treated with IL-23 inhibitors (EAIR, 3.8 per 1000 PY), and two patients treated with IL-12/23 inhibitors (EAIR, 1.8 per 1000 PY). No patients treated with IL-17 inhibitors reported MACE during the treatment.

## 4. Discussion

The results of our study show minimal to low incidences of new-onset IBD, oral/GI candidiasis, herpes zoster, pulmonary tuberculosis, and MACEs in biologically treated patients with moderate-to-severe psoriasis. Although oral/GI candidiasis was reported in patients treated with IL-17 inhibitors (EAIR of 5.3 per 1000 PY), the incidence was comparable to that in those treated with IL-23 inhibitors. Herpes zoster was the most common adverse event and was highly reported in patients treated with TNF-α inhibitors, followed by those treated with IL-17, IL-23, and IL-12/23 inhibitors.

IBD is a chronic autoimmune condition of the large intestine that encompasses ulcerative colitis and Crohn’s disease. Disease onset usually occurs in the second to fourth decade of life [11]. Patients with psoriasis are at high risk of having IBD, especially young patients and those with severe psoriasis [12]. Although evidence from mouse models, human tissue analysis, and genetic studies strongly implicates the Th17 pathway in the pathogenesis of Crohn’s disease, a phase II trial failed to demonstrate the efficacy of secukinumab in patients with moderate-to-severe Crohn’s disease [13]. Unexpectedly, higher rates of adverse events, especially disease aggravation, were noted in those treated with secukinumab than in those treated with placebo.

Current guidelines recommend avoiding the administration of IL-17 inhibitors in patients with psoriasis who have a personal history of or active IBD. Our finding was consistent with this recommendation in that IL-17 inhibitors were not prescribed to patients with psoriasis and IBD [7]. However, whether the blockade of IL-17 is associated with the onset of IBD in patients with psoriasis remains unclear, especially in young patients in whom IBD symptoms have not yet manifested. In a pooled analysis of clinical trials of IL-17 inhibitors, the association between secukinumab treatment and new-onset IBD was not observed (EAIR was 0.01–0.33/100 PY), which was comparable to the findings obtained for etanercept treatment [14,15]. Further, a postmarketing surveillance analysis showed that new-onset IBD was uncommon following IL-17 blockade (EAIR of 0.2 per 100 PY) [14]. The present study also supports that the risk of new-onset IBD in patients with psoriasis receiving IL-17 inhibitors is extremely low.

Both human and mouse studies have revealed that IL-17A plays a role in the mucocutaneous defense against *Candida albicans* [16,17,18]. In a pooled analysis of clinical trials, the overall EAIR of *Candida* infections was higher in secukinumab-treated patients (2.2/100 PY) than in either etanercept- or placebo-treated patients and was dose-dependent [14]. Strong associations between IL-17 inhibitors and oropharyngeal and esophageal candidiasis (odds ratios, 19.18 and 21.20, respectively) were found in real-world observational studies, with the associated risks being four- to ten-fold higher than those associated with TNF-α inhibitors [19]. Our results also showed that the incidence of oral/GI candidiasis was higher in the patients receiving IL-17 inhibitors than in those receiving TNF-α or IL-12/23 inhibitors.

We found a comparable incidence of oral/GI candidiasis in patients with psoriasis treated using IL-17 and IL-23 inhibitors. In two phase III clinical trials in which patients with psoriasis were randomly allocated to receive IL-23 inhibitors or secukinumab, the incidences of *Candida* infections were comparable between the two groups (risankizumab, 1.83% vs. secukinumab, 2.45%; guselkumab, 2% vs. secukinumab, 6%) [20,21]. Not only IL-17-deficient but also IL-23-deficient mice exhibit a suppressed host defense against cutaneous candidiasis compared with IL-12- or IL-22-deficient mice [17]. Innate and adaptive immune response defects in *Candida albicans* were reported in patients with psoriasis who had IL-12/23 signaling blockages [22]. Considering that currently available real-world data on the safety of IL-23 inhibitors is limited and involves only a small number of patients, the risk of oral/GI candidiasis in patients with psoriasis who use IL-23 inhibitors is unclear and needs to be evaluated in real-world settings [23].

Because biological therapies inhibit important cytokines in innate and adaptive immunity, concerns regarding the development of opportunistic infections, including tuberculosis activation and herpes zoster, have existed. Th1 cells and cytokines, such as IFN-γ, IL-12, and TNF-α, play a role in the host defense mechanism against *Mycoplasma tuberculosis* [24]. TNF-α inhibitors that suppress Th1 responses have been recognized to increase the risk of tuberculosis activation since their introduction in the treatment of rheumatoid arthritis and ankylosing spondylitis [25]. Thus, it is highly recommended to conduct screening tests (interferon-γ release assay and chest X-ray) and annual screening for latent tuberculosis [7,26]. For patients with latent tuberculosis, 6 to 9 months of chemoprophylaxis with isoniazid or rifampicin monotherapy is recommended from 3 weeks before the initiation of biologic treatment [26].

However, the role of Th17 cells in the host defense mechanism against *Mycobacterium tuberculosis* is controversial. Although preclinical studies have demonstrated that IL-17A induces neutrophil recruitment and a local inflammatory response via cytokine and chemokine secretion from tissue-resident cells, the functions of IL-23 and IL-17 in tuberculosis appear to be more subtle than those of Th1 cytokines [27]. Contrary to the many cases of tuberculosis reactivation during treatment with TNF-α inhibitors and a few cases associated with IL-12/23 inhibitors, there have been no reported cases in patients exposed to IL-17 or IL-23 inhibitors both in clinical and real-world settings, which was consistent with our results [14,28,29,30,31]. Based on these clinical data, some authors are skeptical about annual screening in asymptomatic patients receiving IL-17 and IL-23 inhibitors [32].

The risk of opportunistic infection by the varicella zoster virus increases in biologic therapy-receiving patients with psoriasis. Regarding TNF-α inhibitors, a significant elevation in the risk of herpes zoster was not found in patients with psoriasis treated with adalimumab and etanercept [33]. Although infliximab and ustekinumab seem to exhibit higher risks than adalimumab, no statistical significance was observed in a meta-analysis [34]. Regarding IL-17 and IL-23 inhibitors, a recent population-based observational study found a lower risk of herpes zoster associated with them than with TNF-α inhibitors, with hazard ratios of 0.58 and 0.79, respectively [35]. The results of our study were consistent with those of previous studies, showing a higher incidence of herpes zoster associated with TNF-α inhibitor use than with the use of IL-17 and IL-23 inhibitors. The incidences of herpes zoster in patients with psoriasis treated using IL-17 and IL-23 inhibitors were comparable to those in the general Korean population (13.3 per 1000 PY) [36].

Psoriasis is a risk factor for myocardial infarction, coronary artery disease, and stroke, especially in young patients or patients with severe disease [37,38]. IL-17 could play a role in the development of CVD, as indicated by the high mortality rate and recurrence of acute myocardial infarction in patients with low serum IL-17 levels [39]. However, whether IL-17 blockade has a beneficial effect on CVD risk in patients with psoriasis remains uncertain. Coronary computed tomography and angiography-based evaluations in patients with moderate-to-severe psoriasis showed that the use of biological therapies, including IL-17 inhibitors, was associated with a reduction in high risk coronary plaque phenotypes [40]. In a post hoc analysis of pooled data from phase III/IV secukinumab studies in patients with psoriasis and PsA, reductions in inflammatory biomarker levels predictive of CVD and CVD-related mortality, C-reactive protein level, and neutrophil-to-lymphocyte ratio appeared as early as week 12 and were sustained over 52 weeks [41]. Endothelial function measured by flow-mediated dilation showed a significant improvement in patients with moderate-to-severe plaque psoriasis who were treated with secukinumab for 52 weeks [9]. In contrast, low plasma IL-17A levels were associated with an increased incidence of CVD in patients with moderate-to-severe psoriasis [42]. There was no beneficial effect on aortic vascular inflammation, which is an imaging biomarker of CVD risk, in patients treated with secukinumab [43]. A significant association between IL-17A blockade and incidences of MACEs was not found in clinical trials [44].

The present study has several limitations. Because there were only a small number of patients experiencing adverse events, a statistical analysis between the groups was not feasible. OMOP-CDM does not provide detailed information, including the time interval between the initiation of biological therapy and the occurrence of adverse events, the severity of adverse events, whether biological therapy was stopped owing to adverse events, a detailed medical history, and the age of onset of psoriasis. As most of the patients were Koreans, caution is required when interpreting the results for other ethnicities. A higher proportion of comorbid PsA in patients treated with TNF-α inhibitors than the other biologics could act as a predisposition to the high incidence of herpes zoster and MACEs. However, a subanalysis of the patients treated with TNF-α inhibitors showed a relatively lower incidence of herpes zoster (16.06 vs. 17.86 per 1000 PY) and MACEs (3.95 vs. 7.02 per 1000 PY) in patients with PsA compared with those without PsA.

Nevertheless, our study provides real-world safety data on currently available biologics from the hitherto largest Korean cohort of patients with moderate-to-severe psoriasis. Incidence was expressed as EAIR, which adjusts for differences in the duration of drug exposure, and not as the crude incidence rate, to enhance the robustness of the results. We primarily focused on adverse events that are of interest to dermatologists to provide a rationale for selecting biologics in the treatment of moderate-to-severe psoriasis.

## 5. Conclusions

The present study showed favorable safety profiles of currently available biologics in Korean patients with moderate-to-severe psoriasis. Compared with IL-23 blockade, IL-17 blockade appears to be associated with minimal risks of new-onset IBD, pulmonary tuberculosis, and MACEs and comparable risks of oral/GI candidiasis.

## Figures and Tables

**Table 1:** Baseline demographic characteristics of the patients.

	All Patients (*n* = 2085)	TNF-α Inhibitor Group (*n* = 463)	IL-12/23 Inhibitor Group (*n* = 540)	IL-17 Inhibitor Group (*n* = 635)	IL-23 Inhibitor Group (*n* = 447)
Age, years, mean ± SD	45.6 ± 13.4	42.1 ± 13.1	44.8 ± 13.2	46.7 ± 13.3	48.7 ± 13.9
Sex, *n* (%)					
Male	1356 (65)	280 (60.5)	353 (65.4)	425 (66.9)	298 (66.7)
Female	729 (35)	183 (39.5)	187 (34.6)	210 (33.1)	149 (33.3)
Previous exposure to biologics, *n* (%)	496 (23.8)	70 (15.1)	95 (17.6)	189 (29.8)	142 (31.8)
Presence of PsA, *n* (%)	395 (18.9)	184 (39.7)	70 (13.0)	113 (17.8)	28 (6.3)
Comorbidities, *n* (%)					
Hypertension	207 (9.9)	48 (10.4)	45 (8.3)	70 (11.0)	44 (9.8)
T2DM	138 (6.6)	28 (6.0)	31 (5.7)	44 (6.9)	35 (7.8)
Hyperlipidemia	162 (7.8)	35 (7.6)	46 (8.5)	50 (7.9)	31 (6.9)
IBD	23 (1.1)	17 (3.7)	5 (0.9)	0 (0.0)	1 (0.2)
Hx of MACEs, *n* (%)	30 (1.4)	7 (1.5)	7 (1.3)	9 (1.4)	7 (1.4)

IBD, inflammatory bowel disease; MACEs, major cardiovascular events; PsA, psoriatic arthritis; T2DM, type 2 diabetes mellitus.

**Table 2:** The number of incident cases and exposure-adjusted incidence rates of adverse events in biologic-treated patients with moderate-to-severe psoriasis.

	TNF-α Inhibitor Group (*n* = 463)			IL-12/23 Inhibitor Group (*n* = 540)			IL-17 Inhibitor Group (*n* = 635)			IL-23 Inhibitor Group (*n* = 447)		
	TAR (PY)	Cases (*n*)	EAIR (per 1000 PY)	TAR (PY)	Cases (*n*)	EAIR (per 1000 PY)	TAR (PY)	Cases (*n*)	EAIR (per 1000 PY)	TAR (PY)	Cases (*n*)	EAIR (per 1000 PY)
New-onset IBD	496	0	0	1154	0	0	774	0	0	537	0	0
Oral/GI candidiasis	542	0	0	1154	1	0.9	761	4	5.3	536	3	5.6
Pulmonary tuberculosis	539	1	1.9	1153	1	0.9	767	0	0	537	0	0
Herpes zoster	529	9	17.0	1122	3	2.7	752	10	13.3	512	4	7.8
MACEs	538	3	5.6	1141	2	1.8	764	0	0	528	2	3.8

EAIR, exposure-adjusted incidence rate; IBD, inflammatory bowel disease; MACEs, major cardiovascular events; TAR, time at risk.

## Data Availability

The data presented in this study are available on request from the corresponding author.
